# Angiotensin II Attenuates the Bioactivities of Human Endothelial Progenitor Cells via Downregulation of *β*2-Adrenergic Receptor

**DOI:** 10.1155/2018/7453161

**Published:** 2018-10-29

**Authors:** Seon Jin Lee, Da Yeon Kim, Jisoo Yun, Sung Hyun Choi, Seok Yun Jung, Songhwa Kang, Ji Hye Park, Yeon Ju Kim, Jong Seong Ha, Seung Taek Ji, Woong Bi Jang, Dong Hyung Lee, Dongjun Lee, Sang-Mo Kwon

**Affiliations:** ^1^Department of Physiology, Laboratory for Vascular Medicine and Stem Cell Biology, Convergence Stem Cell Research Center, Medical Research Institute, Pusan National University School of Medicine, Yangsan 50612, Republic of Korea; ^2^Cellular Therapeutics Development Team, Bio Center, Institute of Daewoong Life Science, Department of R&D, Daewoong Pharmaceutical Co., Ltd, Yongin-si, Gyeonggi-do 17028, Republic of Korea; ^3^Department of Obstetrics and Gynecology, Pusan National University, School of Medicine, Busan 50612, Republic of Korea; ^4^Department of Medical Science, Pusan National University School of Medicine, Yangsan 50612, Republic of Korea

## Abstract

Cross talks between the renin-angiotensin system (RAS), sympathetic nervous system, and vascular homeostasis are tightly coordinated in hypertension. Angiotensin II (Ang II), a key factor in RAS, when abnormally activated, affects the number and bioactivity of circulating human endothelial progenitor cells (hEPCs) in hypertensive patients. In this study, we investigated how the augmentation of Ang II regulates adrenergic receptor-mediated signaling and angiogenic bioactivities of hEPCs. Interestingly, the short-term treatment of hEPCs with Ang II drastically attenuated the expression of beta-2 adrenergic receptor (ADRB2), but did not alter the expression of beta-1 adrenergic receptor (ADRB1) and Ang II type 1 receptor (AT1R). EPC functional assay clearly demonstrated that the treatment with ADRB2 agonists significantly increased EPC bioactivities including cell proliferation, migration, and tube formation abilities. However, EPC bioactivities were decreased dramatically when treated with Ang II. Importantly, the attenuation of EPC bioactivities by Ang II was restored by treatment with an AT1R antagonist (telmisartan; TERT). We found that AT1R binds to ADRB2 in physiological conditions, but this binding is significantly decreased in the presence of Ang II. Furthermore, TERT, an Ang II-AT1R interaction blocker, restored the interaction between AT1R and ADRB2, suggesting that Ang II might induce the dysfunction of EPCs via downregulation of ADRB2, and an AT1R blocker could prevent Ang II-mediated ADRB2 depletion in EPCs. Taken together, our report provides novel insights into potential therapeutic approaches for hypertension-related cardiovascular diseases.

## 1. Introduction

Hypertension is a progressive disease involving abnormalities in the renin-angiotensin-sympathetic interactions [1]. Both the renin-angiotensin system (RAS) and the adrenergic nervous system operate mutually to maintain blood pressure homeostasis [2]. Multiple reports suggest that hyperactivity of these systems has pathophysiological relevance, such as causing cardiorenal disease and hypertension [3, 4]. Pathological stimuli, including cardiorenal disease, hypertension, and stroke, are also involved in the development of abnormal vessel formation [5]. Human endothelial progenitor cells (hEPCs) are used in cell therapy to repair tissue and induce vascular regeneration [6]. These EPCs mobilize into ischemic sites and aid neovessel formation [7, 8]. However, angiotensin II (Ang II) and other cytokines reduce the number and bioactivities of EPCs in patients [9–11]. Ang II, a known cause of hypertension [12], affects multiple cells including CD34-positive progenitor cells and the hematopoietic precursor of dendritic cells through the RAS pathway [13, 14].

Multiple small-molecule inhibitors have been used to prevent endothelial dysfunction that occurs in response to Ang II [15]. Angiotensin II type 1 receptor (AT1R) blockers [16], angiotensin II-converting enzyme inhibitors [17], and *β*-adrenergic blockers [18] are commonly used to treat hypertension. A recent study demonstrated a functional and physiological interaction between AT1R and *β*-adrenergic receptor (*β*AR) and the transinhibitory effects of angiotensin receptor blockers and *β*-adrenergic receptor blockers [19]. Moreover, it has been shown that *β*AR activation is required for the maintenance of renin synthesis and release [20]. Toth et al. also reported that AT1R is directly interacted with *β*2-adrenergic receptor (ADRB2) via heterodimerization [21]. However, the effect of interaction between AT1R and ADRB2 on endothelial dysfunction has not been elucidated.

The current study was designed to investigate if augmented Ang II levels, as seen in hypertensive patients, act as a negative regulator of *β*AR in EPCs. Here, we provide evidence to demonstrate that augmented Ang II affects EPC bioactivities and ADRB2 expression in EPCs. In clinical settings, we report an important observation that inhibition of Ang II using telmisartan (an AT1R blocker) reversed the negative effect on EPC bioactivities caused by Ang II, by restoring complex formation of AT1R and ADRB2. Thus, these findings provide novel insights for the therapy of vascular disease such as hypertension.

## 2. Materials and Methods

### 2.1. Human Endothelial Progenitor Cells

Human umbilical cord blood was supplied by Pusan National University Yangsan Hospital (PNUYH). The separation of human EPCs and all other experiments were approved by the International Review Board (IRB) of Pusan National University Hospital (IRB No. 05-2017-053). Mononuclear cells (MNCs) were isolated from cord blood by density gradient centrifugation with Ficoll separating solution (GE Healthcare, UK). The MNCs were cultured in Endothelial Cell Growth Medium-2 BulletKit medium (EGM-2, Lonza, USA) supplemented with 5% fetal bovine serum (FBS), hEGF, bFGF, hVEGF, hIGF-1, ascorbic acid, GA-1000, and 1x penicillin–streptomycin (Welgene, Korea). After 5 days, nonadherent cells were discarded, and the attached cells were cultured further with fresh EGM-2. Spindle-shaped colonies appeared between days 5 and 21; subsequently, isolated EPCs were verified for their ability for acetylated low-density lipoprotein (Ac-LDL) uptake (Dil-acLDL, Invitrogen, USA). EPCs were used at passages 7–9 for all experiments.

### 2.2. Cell Viability Assay

The effects of Ang II and TERT (Sigma-Aldrich, USA) on the viability of EPCs were evaluated by WST-1 assay (EZ-Cytox, Daeil Lab, Korea). EPCs (1 × 10^5^) in EGM-2 were seeded into 96-well plates and incubated. After 12 h, the medium was replaced with EGM-2 containing 1% FBS, and incubation was continued for 12 h. Following this, serially diluted Ang II (0, 10 nM, 100 nM, 1 *μ*M, and 10 *μ*M) was added to the actively growing cells. After 24 h of incubation, WST solution was added to each well, and the absorbance was detected at 450 nm at 25°C.

### 2.3. Western Blot Analysis and Immunoprecipitation

Western blot analysis was performed to assess the cellular expression levels of ADRB1, ADRB2, AT1R, and *β*-actin in EPCs. Rabbit polyclonal anti-ADRB1 (1 : 1000, Abcam, UK), rabbit polyclonal anti-ADRB2 (1 : 1000, Abcam), mouse monoclonal anti-AT1R (1 : 1000, Abcam), and mouse monoclonal anti-*β*-actin (1 : 5000, Santa Cruz Biotechnology, USA) were used. EPCs were first treated with Ang II or TERT, following which the total cellular proteins were extracted using RIPA lysis buffer (ELPIS BIOTECH, Korea). The proteins (15–20 *μ*g) were then separated by SDS-PAGE and transferred to polyvinylidene fluoride membranes (Millipore, USA). The membranes were blocked with 5% skim milk for 1 h at 25°C and incubated overnight with the specific primary antibodies mentioned above, at 4°C. For the immunoprecipitation assay, cell lysates (1 mg) were incubated with anti-AT1R antibody (Abcam) and then with Protein A/G PLUS-Agarose beads (Santa Cruz Biotechnology). The eluents (25 *μ*l) were separated by SDS-PAGE and detected with ADRB2 antibody (Abcam). Quantitative analysis of the band was performed using ImageJ software, and the Western blot result for each studied protein was normalized with that for *β*-actin.

### 2.4. Immunocytochemistry

EPCs were seeded at a density of 3 × 10^4^ cells/ml on a 1% gelatin-coated cover glass. After 24 h, the cells were exposed to serum starvation for 12 h prior to treatment with Ang II (100 nM). After 24 h of Ang II treatment, the cells were fixed with 2% paraformaldehyde for 20 min and then permeabilized with 0.3% PBST for 10 min at 25°C. Nonspecific binding was blocked with a blocking solution (5% normal goat serum and 0.3% Triton X-100 in PBS) for 1 h at 25°C. Then, the cells were stained overnight with rabbit polyclonal anti-ADRB1 (1 : 200, Abcam), rabbit polyclonal anti-ADRB2 (1 : 200, Abcam), and mouse monoclonal anti-AT1R (1 : 50, Abcam) at 4°C. Thereafter, the cells were washed and probed with fluorescent dye-conjugated secondary antibodies (1 : 400, rabbit polyclonal Alexa Fluor 594 or mouse monoclonal Alexa Fluor 488, Life Technologies, USA) against the primary antibody for 1 h at 25°C. The nuclei were stained with 4′,6-diamidino-2-phenylindole dihydrochloride (DAPI; Sigma-Aldrich), and the cover glass was mounted using ProLong Diamond Antifade reagent (Life Technologies). Fluorescent images were obtained using a confocal microscope (Olympus, Japan).

### 2.5. Tube Formation Assay

Vascular network formation ability was evaluated using Matrigel assays. EPCs were seeded at a density of 1 × 10^4^ cells on Matrigel (BD Biosciences, USA) in a 96-well plate. Each group of serum-starved, Ang II-induced, or DMSO or AT1R blocker (TERT) plus Ang II-induced EPCs was coplated in 100 *μ*l of various conditioning media: plain EGM-2, EGM-2 with 100 nM (−)-isoproterenol hydrochloride (ISO; Sigma-Aldrich), or EGM-2 with 100 nM formoterol fumarate dihydrate (Formo; Sigma-Aldrich). The cells were incubated at 37°C in a 5% CO_2_ incubator for 6 h. The number of branching points was calculated using ImageJ software.

### 2.6. Migration Assay

To examine the effect of Ang II treatment or the ADRB2 agonist on the cell wound-healing potential, EPCs were seeded in 6-well plates at a density of 1 × 10^6^ cells/well. At 24 h after seeding, the cells were serum-starved for 12 h, following which a straight scratch was made across the cell lawn using a pipette tip. Then, the cells were treated with 100 nM ISO, 100 nM Formo, or 100 nM Ang II or cotreated with Ang II and ISO, or Ang II and Formo, and incubated at 37°C for 6 h. The wound-healing capacity was analyzed with ImageJ software, which calculates the percentage of the closure area.

### 2.7. Cell Cycle Assay

Cell cycle progression was examined in Ang II-induced EPCs stained with propidium iodide (PI; Sigma-Aldrich) using fluorescence-activated cell sorting (FACS). Human EPCs were seeded at a density of 1 × 10^6^ cells in a 100 mm culture dish. At 24 h after Ang II treatment, the cells were harvested, washed with FACS buffer (PBS supplemented with 2% FBS and 2 mM EDTA), and fixed with cold 70% ethanol at 4°C for 1 h. After washing with FACS buffer, the cells were incubated with 0.5 mg/ml RNase A (Sigma-Aldrich) at 37°C for 1 h. The cells were then stained with PI solution (at a final concentration of 10 *μ*g/ml) and analyzed with a flow cytometer (BD FACSCanto II).

### 2.8. Statistical Analysis

Data are presented as the mean ± standard error of the mean (SEM). Statistical significance was evaluated with Student's *t*-test. A *P* value of <0.05 was considered statistically significant.

## 3. Results

### 3.1. Effect of Ang II on EPC Cell Viability

**Figure 1:**
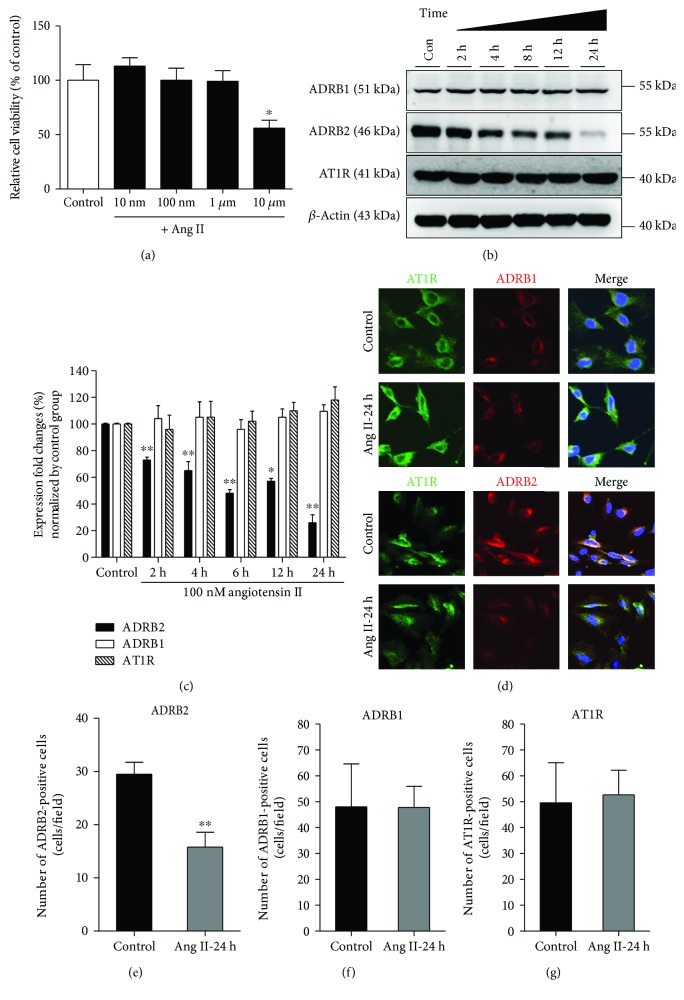
Effect of Ang II on ADRB2 expression and EPC bioactivities. (a) Ang II-induced cytotoxicity in EPCs was measured using WST-1 assay. EPC viability was reduced after treatment with 10 *μ*M Ang II. ^∗^ *P* < 0.05 vs. control. (b). ADRB1, ADRB2, and AT1R levels after time-dependent Ang II treatment were analyzed using Western blotting, and *β*-actin was used as a loading control. (c). Quantitative graph of total protein levels in Ang II-induced EPCs. ^∗^ *P* < 0.01 and ^∗∗^ *P* < 0.001 vs. control. (d) Immunocytochemistry was performed to confirm the expression of ADRB1, ADRB2, and AT1R in the presence of Ang II. Representative cropped images of ADRB1, ADRB2, and AT1R from 20x fluorescent images. (e–g) Quantification of ADRB2-, ADRB1-, and AT1R-positive cells per field. ^∗∗^ *P* < 0.01 vs. control.

To validate the effect of Ang II on EPCs, we first performed the cell viability assay. EPCs were treated with Ang II in a dose-dependent manner (10 nM, 100 nM, 1 *μ*M, and 10 *μ*M) for 24 h (Figure 1(a)). The data showed that the cell viability was reduced to 75% when treated with 10 *μ*M Ang II. Based on this result, 100 nM of Ang II was used for all further experiments.

### 3.2. Ang II Reduces the Expression of ADRB2 in EPCs

Then, we analyzed the effect of Ang II on the expression patterns of ADRB1, ADRB2, and AT1R. EPCs were treated with 100 nM Ang II in a time-dependent manner (0, 2, 4, 8, 12, and 24 h) (Figures 1(b) and 1(c)). Interestingly, treatment with 100 nM Ang II resulted in significant downregulation of ADRB2 in a time-dependent manner. Especially, 24 h after Ang II treatment, ADRB2 was dramatically downregulated. However, Ang II had no effect on ADRB1 or AT1R expression. To confirm the effect of Ang II on ADRB2 downregulation, we analyzed the expression using confocal microscopy. As expected, immunofluorescence data showed decreased expression of ADRB2 in the presence of Ang II, whereas the expression of AT1R and ADRB1 were not affected (Figure 1(d)), which is in conjunction with our immunoblotting data. Quantification data also indicated that ADRB2 expression was decreased to 55% when treated with Ang II, but the expressions of ADRB1 and AT1R were not altered (Figures 1(e)–1(g)). Taken together, this data indicates that Ang II treatment downregulates the expression of ADRB2 in EPCs.

### 3.3. ADRB2 Agonists Increase the EPC Bioactivities

**Figure 2:**
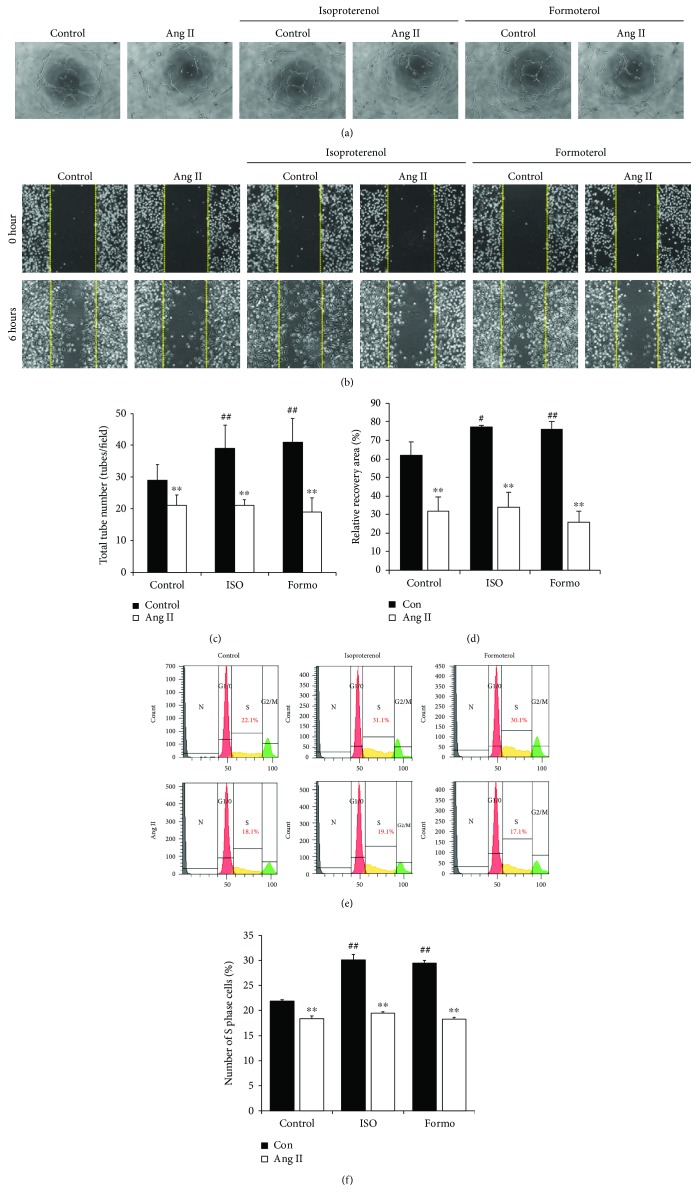
ADRB2 agonists stimulate EPC bioactivities. (a) Human EPCs were pretreated with Ang II, and the effects of ADRB2 agonists (isoproterenol and formoterol) were analyzed for test tube formation ability. (b) Migration ability was estimated by a scratch wound healing assay. Each group of EPCs, control cells, or Ang II-treated cells was seeded, followed by treatment with media containing ADRB2 agonists. (c) Quantification of total tube number was performed using ImageJ software. All experiments were performed in triplicates. ^∗∗^ *P* < 0.01 vs. control, and ^##^ *P* < 0.01 vs. negative control. (d) Quantification of migrated cells was performed using ImageJ software. Recovery area was analyzed and presented. ^∗∗^ *P* < 0.01 vs. control; ^#^ *P* < 0.05 and ^##^ *P* < 0.01 vs. negative control. (e) Propidium iodide (PI) staining of DNA was detected by flow cytometry of EPCs treated with 100 nM Ang II. (f) Graph of the proportion of cells in the S phase for each group as measured by PI staining. ^∗∗^ *P* < 0.01 vs. control, and ^##^ *P* < 0.01 vs. negative control.

To confirm whether the Ang II-induced ADRB2 downregulation influenced EPCs bioactivities, we treated EPCs with the ADRB2 agonists, namely, ISO, a nonselective agonist, and Formo, a selective agonist of ADRB2. As shown in Figures 2(a) and 2(c), treatment with Ang II attenuated the ability of EPCs to form tubes when compared to the control EPCs, whereas ADRB2 agonist treatment led to a marked increase in tube formation ability when compared to the untreated control. However, ADRB2 agonists had no effect on tube formation ability in Ang II-treated EPCs. Moreover, data from the migration assay showed a similar pattern to the results from the tube formation assay. Migratory capacity was attenuated during Ang II treatment, whereas stimulation of ADRB2 using both agonists accelerated migration (Figures 2(b) and 2(d)). However, stimulation of ADRB2 by agonists could not rescue the reduced migratory capacity in Ang II-treated EPCs. To further verify the effect of the ADRB2 agonist on Ang II-treated EPCs, we analyzed the cell cycle progression using PI staining. As shown in Figures 2(e) and 2(f), the percent of S-phase cells in the control, ISO-, and Formo-treated groups were 22.1%, 31.1%, and 30.1%, respectively, indicating that EPC proliferation was significantly increased by ADRB2 agonists. In contrast, Ang II treatment resulted in a significant decrease in cell proliferation ability. However, no significant change was observed after ADRB2 agonist treatment in Ang II-treated EPCs. These results suggest that treatment with Ang II attenuates EPC bioactivities, and treatment of ADRB2 agonists accelerates EPC bioactivities.

### 3.4. An AT1R Blocker Rescues Ang II-Induced ADRB2 Depletion in EPCs

**Figure 3:**
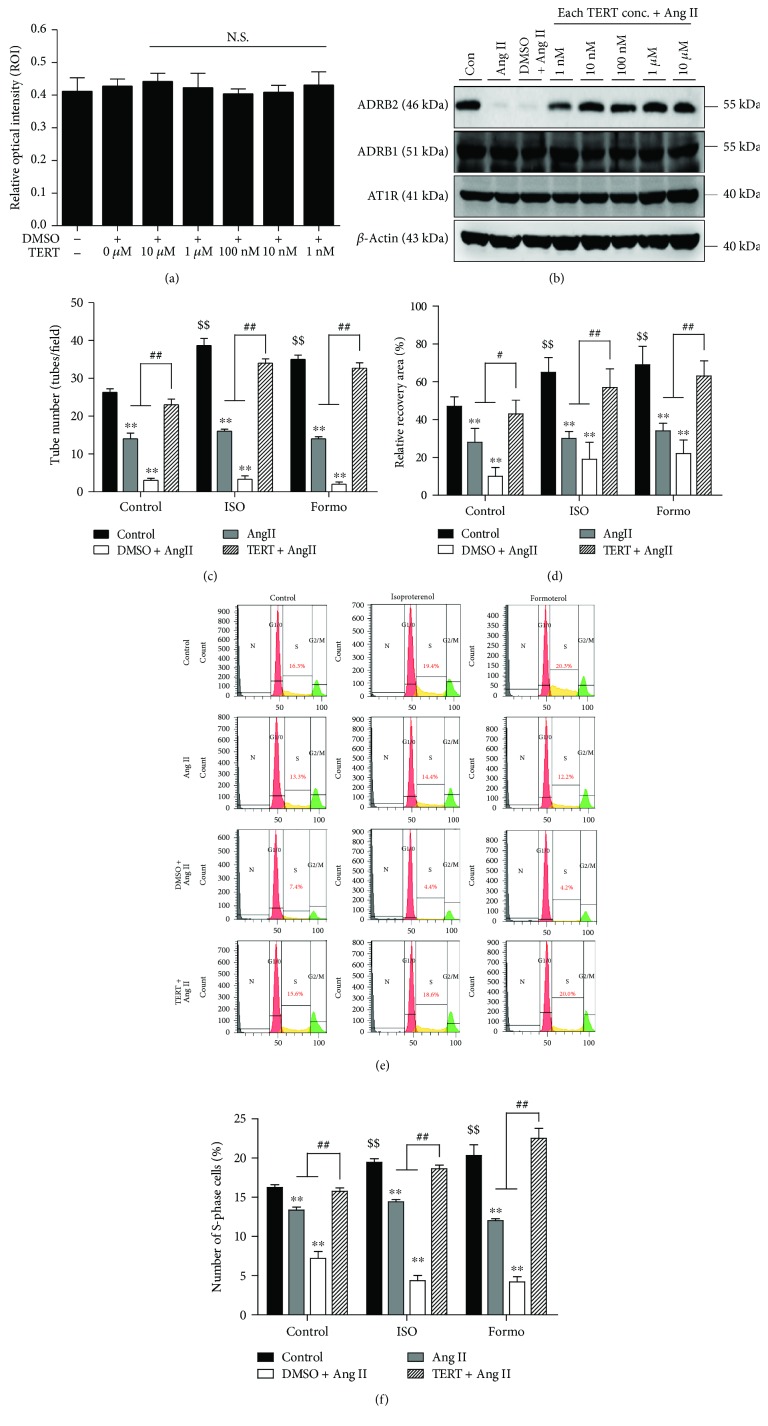
Effects of AT1R blocker, telmisartan (TERT), on EPCs. (a) Cell viability assay upon treatment with TERT, an AT1R blocker, using the WST-1 assay. NS = not significant. (b) Ang II- and TERT-treated EPCs were harvested, and the expression of ADRB1, ADRB2, and AT1R was analyzed using Western blotting. Expression of ADRB2 was decreased following treatment of EPCs with Ang II, whereas protein levels of ADRB2 were restored in AT1R-blocked cells. (c) Human EPCs were pretreated with an AT1R blocker and incubated with Ang II for 24 h. Each group of cells was seeded onto Matrigel GFR with or without ADRB2 agonists. Quantitative graph of the tube formation data. The tube number was measured using ImageJ software. ^∗∗^ *P* < 0.01 vs. control, ^$$^ *P* < 0.01 vs. negative control, and ^##^ *P* < 0.01 vs. Ang II or DMSO plus Ang II. (d) Migration ability was examined using the scratch wound healing assay. Each group of EPCs was seeded, and migratory capacity was observed for 6 h. Quantitative graph of migration assay results. All experiments were performed in triplicates at least. ^∗∗^ *P* < 0.01 vs. control, ^$$^ *P* < 0.01 vs. negative control, and ^#^ *P* < 0.05 and ^##^ *P* < 0.01 vs. Ang II or DMSO plus Ang II. (e, f) PI staining of DNA in EPCs detected by flow cytometry. Graph of the percentage of S-phase cells in each group measured by PI staining. ^∗∗^ *P* < 0.01 vs. control, ^$$^ *P* < 0.01 vs. negative control, and ^##^ *P* < 0.01 vs. Ang II or DMSO plus Ang II.

It is known that the effects of Ang II on EPCs are mediated by its interactions with AT1 and AT2 receptors [22]. Wassmann et al. reported that the Ang II-induced detrimental effects originate from AT1R receptor signaling in EPCs [23]. To study the restorative effect of the AT1R blocker on Ang II-induced ADRB2 depletion, we added TERT to EPCs following Ang II treatment. The cell viability assay was performed to analyze the cytotoxicity of TERT on EPCs, and no cytotoxic effects were observed (Figure 3(a)). Next, we analyzed the restorative effect of TERT on ADRB2 expression, which was depleted on Ang II treatment. As shown in Figure 3(b), Ang II treatment reduced the expression of ADRB2 in EPCs; however, pretreatment with TERT significantly abolished Ang II-induced ADRB2 depletion. Further, TERT had no effect on ADRB1 or AT1R expression levels. These results suggest that downregulation of ADRB2, which is mediated by Ang II treatment, could be restored by treatment with the AT1R blocker.

### 3.5. An AT1R Blocker Improves the Function of EPCs against Ang II-Mediated ADRB2 Downregulation

To test whether TERT could rescue the ADRB2 agonist effect during Ang II treatment, we evaluated the EPC bioactivities with TERT and ADRB2 agonist treatments. As expected, pretreatment with TERT before Ang II treatment resulted in a marked increase in vascular network formation ability compared to treatment with Ang II alone (Figure 3(c) and Supplementary Figure 1). In the presence of TERT, ADRB2 agonists restored the tube formation ability in Ang II-treated EPCs. Similar to results from the tube formation assay, cotreatment with TERT and ADRB2 agonists restored the migratory capacity of EPC (Figure 3(d) and Supplementary Figure 2). EPC proliferation data also showed that TERT abrogates the inhibitory effect of Ang II. Ang II-induced decreased S-phase cells were restored by TERT, suggesting a significant increase in the proliferation rate of EPCs pretreated with TERT after ADRB2 agonist stimulation (Figures 3(e) and 3(f)). These results indicate that inhibition of Ang II using TERT enhances ADRB2 stimulation and restores the Ang II-induced decrease in EPC bioactivities via regulation of ADRB2 expression.

### 3.6. Interaction between AT1R and ADRB2 Is Regulated by Ang II Treatment

**Figure 4:**
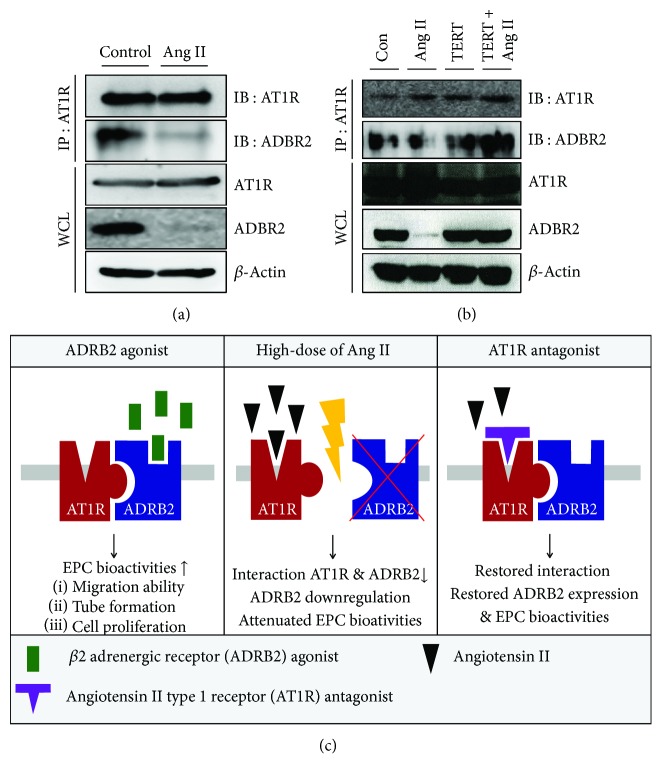
The interaction between AT1R and ADRB2 is attenuated by Ang II. (a) Association between AT1R and ADRB2 in EPCs was confirmed by immunoprecipitation assay. For endogenous IP, lysate from EPCs was immunoprecipitated using a specific antibody, anti-AT1R, followed by detection with ADRB2 antibody. (b) To confirm the role of Ang II in the interaction between AT1R and ADRB2, EPCs were treated with Ang II or TERT. (c) Proposed working model for the role of ADRB2 in Ang II-induced EPC bioactivities.

To confirm the regulatory mechanism of Ang II-induced ADRB2 depletion, we hypothesized that the interaction between AT1R and ADRB2 is tightly regulated by Ang II. To verify our hypothesis, we performed an endogenous immunoprecipitation assay. As shown in Figure 4(a), AT1R and ADRB2 interact in basal conditions; however, this binding was abolished after Ang II treatment. Further, to test whether Ang II mediated this reduced interaction, EPCs were pretreated with TERT, which resulted in restoration of binding between AT1R and ADRB2 (Figure 4(b)). The proposed working model for the role of ADRB2 is illustrated in Figure 4(c). Taken together, these results suggest that Ang II regulates the interaction between AT1R and ADRB2.

## 4. Discussion

In this study, we showed that increase in Ang II impairs EPC bioactivities in an ADRB2-dependent manner. In summary, we demonstrated that (1) Ang II attenuated EPC bioactivities, such as tube formation ability, migratory capacity, and cell proliferation; (2) Ang II dramatically suppressed the expression of ADRB2 in a dose-dependent manner; (3) stimulation of ADRB2 with agonists (ISO and Formo) stimulated EPC bioactivities; and (4) treatment with TERT, an AT1R blocker, not only restored the expression of ADRB2 but also rescued impaired EPC bioactivities induced by Ang II treatment. In the presence of TERT, ADRB2 agonists reversed the reduced EPC bioactivities when treated with Ang II; (5) the direct interaction between ADRB2 and AT1R is attenuated by Ang II, but significantly restored by TERT.

For the first time, we demonstrated the regulatory effect of Ang II on ADRB2 expression, indicating that Ang II acts as a negative regulator of ADRB2 in EPCs, the proposed working model of which is illustrated in Figure 4(c). Interestingly, Ang II did not alter the expression of either ADRB1 or AT1R, but only ADRB2 expression was significantly decreased by Ang II treatment. These results were confirmed by an immunoprecipitation (IP) assay, which showed that ADRB2 binds to AT1R. This result is correlated with previous report [21]. This interaction is impaired by Ang II treatment and restored by TERT treatment. We postulate two possible reasons to explain the attenuated interaction between AT1R and ADRB2. One possibility is that Ang II directly interferes with the complex formation. Alternatively, the decreased expression of ADRB2 induced by Ang II might lead to decreased binding. To prove this, we need to perform further experiments including posttranslational degradation such as ubiquitin-mediated degradation. Taken together, our results suggest that Ang II induces the dysfunction of EPCs via downregulation of ADRB2 and that Ang II-mediated ADRB2 depletion in EPCs can be prevented by an AT1R blocker.

In Figure 2, we clearly demonstrated that proper stimulation of ADRB2 using agonists (ISO and Formo) increases EPC bioactivities, such as angiogenic potential, migratory capacity, and cell proliferation. Accumulating evidence supports our data, implying that ADRB2 stimulation by agonists leads to a significant increase in the angiogenic potential of EPCs [24]. Iaccarino et al. also reported that increased ADRB2 modulates angiogenesis in response to chronic ischemia in endothelial cells [25]. However, in our study, Ang II-induced EPC dysfunction was not fully rescued by treatment with ADRB2 agonists alone. We hypothesized that Ang II-induced EPC dysfunction occurred due to ADRB2 depletion; however, our results showed that the treatment with ADRB2 agonists is not sufficient to restore the reduction in EPC bioactivities induced by Ang II. We speculated that the protein level of ADRB2 might be too low after Ang II treatment to be activated by the ADRB2 agonist. To confirm our hypothesis, we need to test whether the protein level of ADRB2 is restored in the presence of the ADRB2 agonist. In support of our hypothesis, as shown in Figure 3, TERT restored the expression of ADRB2. Additionally, in the presence of TERT, stimulation of ADRB2 by agonists rescued the Ang II-induced EPC dysfunction. Taken together, these data suggest that ADRB2-related signaling might be closely related to Ang II-induced EPC dysfunction.

According to a previous report, the concentration of Ang II in the arterial blood of hypertensive patients (essential hypertension) was 5.2 ng/100 ml, whereas that in healthy arterial blood was 2.1 ng/100 ml [26]. Because the human vascular network is systemic and complex, there is a limitation to mimicking the Ang II dose of the hypertensive patient into the *in vitro* culture system. Several publications reported the dose of 100 nM Ang II in EPCs [27, 28]. In addition, because our data showed that 100 nM Ang II had no effect on cell viability, we used this dose for all the experiments. In the case of TERT, the dosage for hypertensive patients is 20–160 mg once daily, of which compatibility between clinical data and the *in vitro* culture system is also difficult to attain.

According to our results, Ang II attenuated EPC bioactivities, such as migration and tube formation, which correlated with previous works [10, 29]. However, several reports have suggested that Ang II enhances the EPC angiogenic potential via Flt-1 and KDR expression [30, 31], which is contrary to our results. In neovascularization, Ang II has been shown to stimulate vessel formation via VEGF production in the skeletal myocytes [32] and microvascular endothelium [33], whereas Ang II impaired EPC bioactivities and vascular regeneration [10] and even accelerated EPC senescence. These reports suggest that Ang II has both positive and negative effects on angiogenesis, and it is a critical regulator in vascular homeostasis [34].

Increasing evidence has suggested that Ang II induces EPC senescence. According to a recent report, a hypertensive rat model (designed by releasing Ang II) showed increased EPC senescence, whereas cotreatment with Ang II and valsartan (an AT1R blocker) resulted in delayed senescence [9]. Imanishi et al. also reported that Ang II increased the gp91phox (NOX2) mRNA level, which led to oxidative stress-mediated senescence, whereas treatment with an AT1R blocker attenuated the Ang II-induced gp91phox expression [27]. They also confirmed Ang II-induced senescence through SA-*β*-gal staining and observation of decreased telomerase activity; however, treatment with the AT1R blocker impaired SA-*β*-gal staining and restored the telomerase activity. These results suggested that TERT has the potential to restore Ang II-induced EPC senescence by regulating the interaction between ADRB2 and AT1R.

Small molecules, such as TERT that inhibit the angiotensin signaling pathway, are used in the treatment of cardiovascular disease [35]. A recent study showed that TERT decreases ROS levels in the circulatory system and has more beneficial effects on the cardiovascular system than *β*AR blockers [36]. Based on our data, we propose that Ang II-mediated ADRB2 depletion in EPCs can be prevented by an AT1R blocker, such as TERT. Moreover, TERT rescued the ability of ADRB2 to stimulate EPCs. It may be useful to administer antihypertensive drugs to hypertensive patients in order to prevent adrenergic dysfunction. Our data clearly show that Ang II negatively affects the cross talks of the adrenergic nervous system and vascular circuit in the context of EPC biology by decreasing intracellular activation by ADRB2. Therefore, a clinically applicable AT1R blocker, TERT, can be used to diminish Ang II-mediated ADRB2 depletion. Our results also indicate that this may have great value as a therapeutic approach in the chronic hypertensive condition because ADRB2 enhances the functions of EPCs and therefore plays an important role in endothelial regeneration. Thus, this novel insight of Ang II-mediated ADRB2 depletion in EPCs provides a potential approach in the treatment of hypertension-related cardiovascular diseases.

## 5. Conclusions

In this study, we firstly demonstrated that Ang II-induced EPC dysfunction is caused by ADRB2 depletion. Surprisingly, Ang II dramatically suppressed the expression of ADRB2 in a dose-dependent manner, which means Ang II plays a role of an ADRB2-negative regulator. Treatment of TERT, an AT1R blocker, restored the expression of ADRB2 but also Ang II-induced EPC dysfunction. We also confirmed that the interaction between AT1R and ADRB2 is regulated by Ang II. Together, these results suggested a novel insight into therapy of vascular disease such as hypertension.

## Data Availability

All data used to support the findings of this study are included within the article and supplementary information file.

## Supplementary Materials

Supplementary Materials Supplementary Figure 1S: human EPCs were pretreated with an AT1R blocker and incubated with Ang II for 24 hr. Each group of cells was seeded onto Matrigel GFR with or without ADRB2 agonists. Tube formation was observed during 6 hr. All experiments were performed in at least triplicates. Supplementary Figure 2S: migration ability was examined using the scratch wound healing assay. Each group of EPCs was seeded, and migratory capacity was observed for 6 hr. All experiments were performed in at least triplicates.
