# Supplementary material for: Angiotensin II Attenuates the Bioactivities of Human Endothelial Progenitor Cells via Downregulation of β2-Adrenergic Receptor
Source: Stem Cells Int. 2018 Oct 29;2018:7453161. doi: 10.1155/2018/7453161 (PMC6231359; doi:10.1155/2018/7453161)
Supplement: Supplementary Materials — Supplementary Figure 1S: human EPCs were pretreated with an AT1R blocker and incubated with Ang II for 24 hr. Each group of cells was seeded onto Matrigel GFR with or without ADRB2 agonists. Tube formation was observed during 6 hr. All experiments were performed in at least triplicates. Supplementary Figure 2S: migration ability was examined using the scratch wound healing assay. Each group of EPCs was seeded, and migratory capacity was observed for 6 hr. All experiments were performed in at least triplicates. [file 7453161.f1.pdf]

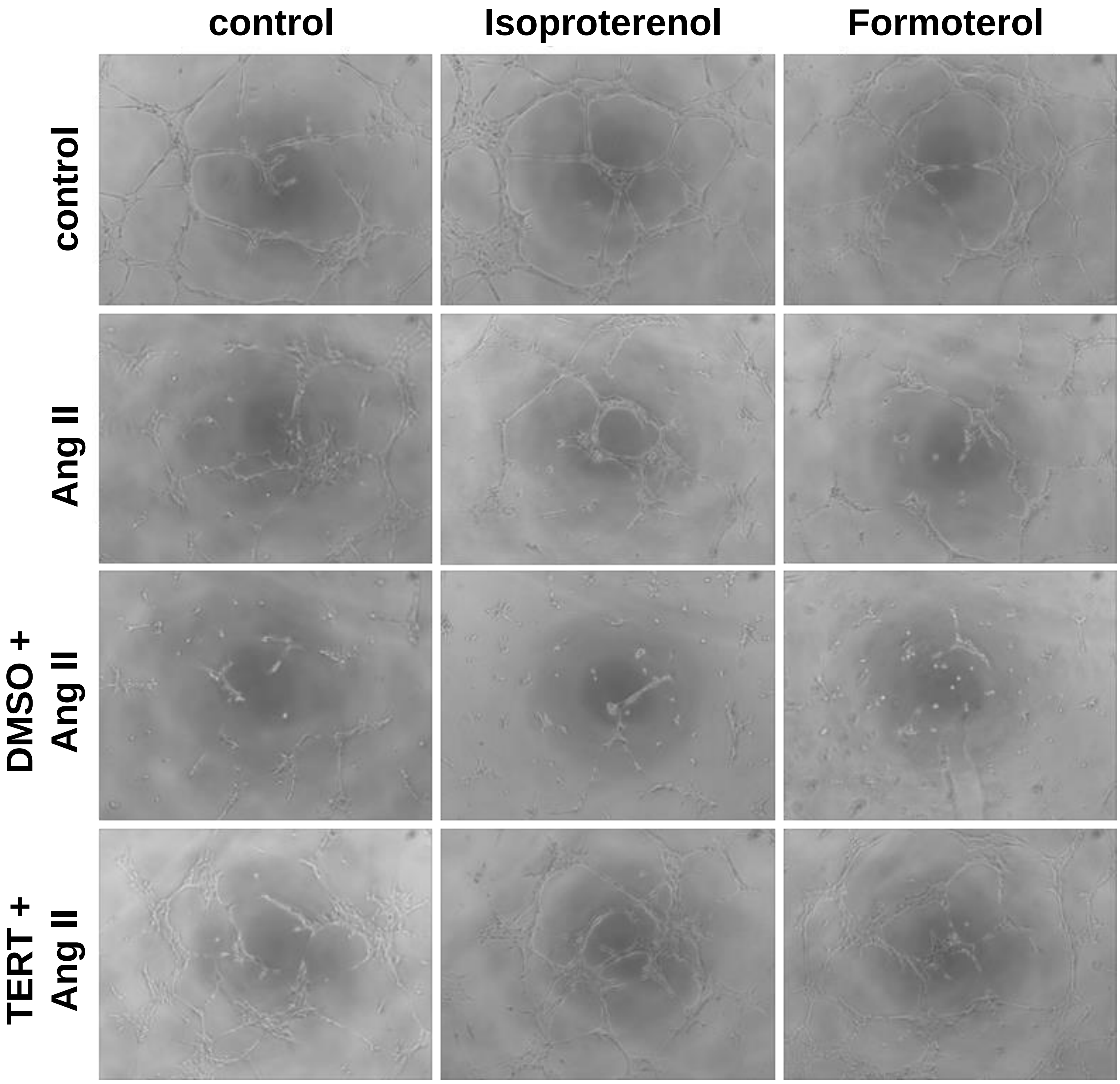

Supplementary Figure 1

Human EPCs were pretreated with an AT1R blocker and incubated with Ang II for 24hr. Each group of cells were seeded onto Matrigel GFR with or without ADRB2 agonists. Tube formation was observed during 6hr. All experiments were performed at least triplicates.

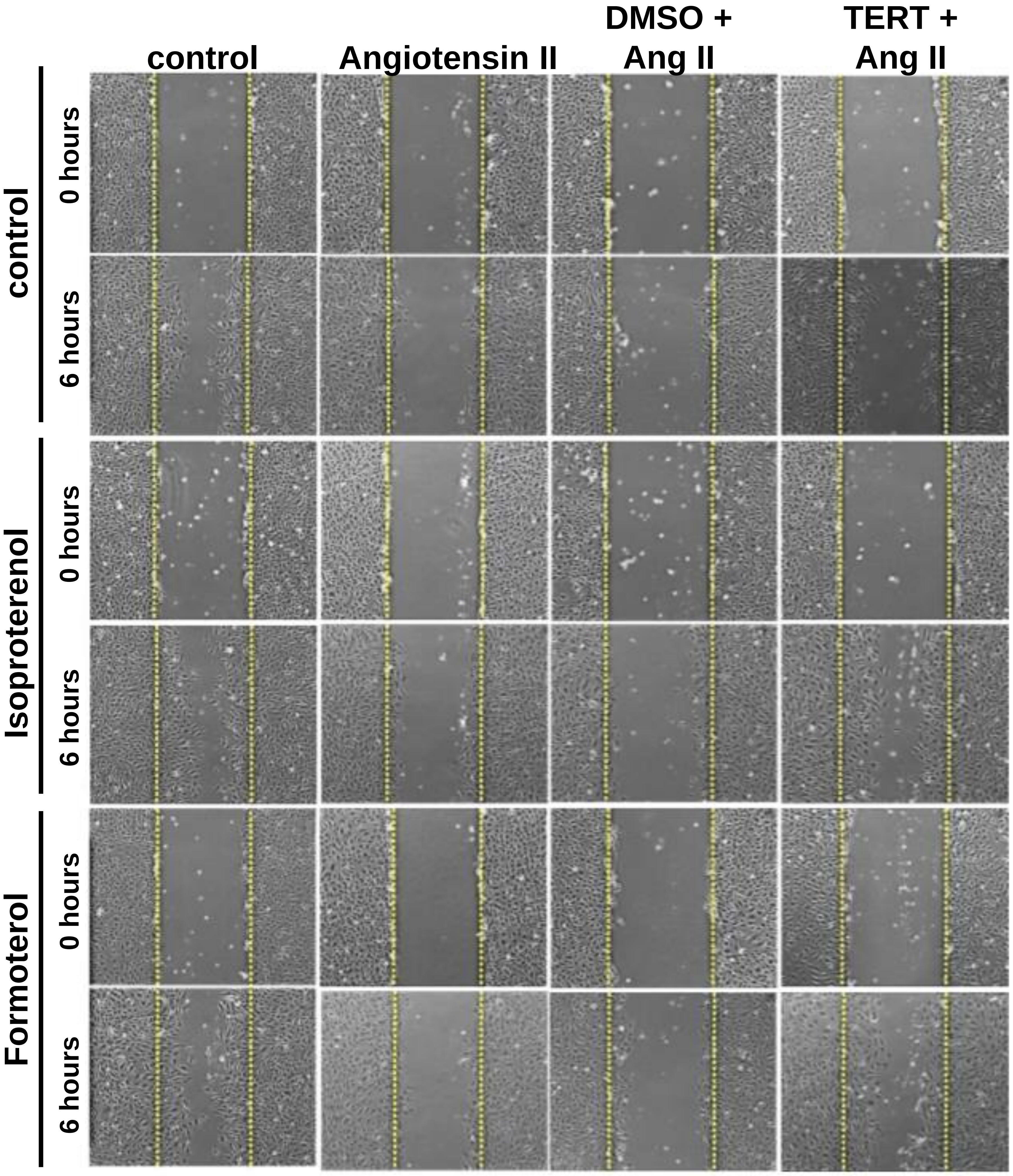

Supplementary Figure 2

Migration ability was examined using the scratch wound healing assay. Each group of EPCs was seeded and observed migratory capacity for 6hr. All experiments were performed at least triplicates.
